# Minimal Residual Disease Detection: Implications for Clinical Diagnosis and Cancer Patient Treatment

**DOI:** 10.1002/mco2.70193

**Published:** 2025-05-15

**Authors:** Meiling Song, Wenjing Pan, Xinjie Yu, Jie Ren, Congli Tang, Zhu Chen, Zhe Wang, Yan Deng, Nongyue He, Hongna Liu, Song Li

**Affiliations:** ^1^ Hunan Key Laboratory of Biomedical Nanomaterials and Devices Hunan University of Technology Zhuzhou China; ^2^ Department of Cell Biology and Genetics School of Basic Medical Sciences Hengyang Medical School University of South China Hengyang China; ^3^ Institute for Future Sciences University of South China Changsha China; ^4^ National Health Commission Key Laboratory of Birth Defect Research and Prevention Changsha China; ^5^ State Key Laboratory of Bioelectronics School of Biological Science and Medical Engineering Southeast University Nanjing China; ^6^ Key Laboratory of Rare Pediatric Diseases Ministry of Education University of South China Hengyang China; ^7^ Guangdong Provincial Hospital of Chinese Medicine & Guangdong Provincial Academy of Chinese Medical Sciences Guangzhou China

**Keywords:** MRD, detection methods, next‐generation sequencing, clinical implication

## Abstract

Minimal residual disease (MRD) serves as a pivotal biomarker for the clinical diagnosis and subsequent treatment of cancer patients. In hematological malignancies, MRD pose an increasingly serious threat to the health of Chinese people. Accurate MRD detection is essential for assessing relapse risk and optimizing therapeutic strategies, yet current methods such as flow cytometry, polymerase chain reaction (PCR), and next‐generation sequencing (NGS) each have distinct limitations, and significant gaps remain in achieving optimal sensitivity and specificity of these technologies. This review provides a comprehensive analysis of MRD detection methods, high‐lighting their clinical implications, including their roles in treatment decision‐making, risk stratification, and patient outcomes. It discusses the strengths and weaknesses of existing techniques and explores emerging technologies that promise enhanced diagnostic precision. Key advancements such as integrating NGS with other methodologies and novel approaches like liquid biopsy and PCR are examined. The review underscores the academic and practical value of early and accurate MRD detection, emphasizing its impact on improving patient management and treatment outcomes. By addressing the limitations of current technologies and exploring future directions, this review aims to advance the field and support personalized medicine approaches to cancer treatment.

## Graphical abstract

Currently, a variety of methods such as flow cytometry, polymerase chain reaction, next‐generation sequencing, and other emerging methods are used for the detection of minimal residual disease, each of which has its own unique strengths and limitations and, more importantly, promising clinical applications.

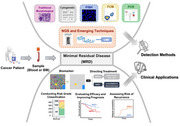

## 1 Introduction

In China, hematological malignancies, including leukemia, lymphoma, and multiple myeloma (MM), represent a significant and growing health threat, with increasing morbidity and mortality rates each year. It is estimated that tens of thousands of patients with these conditions die annually, with the mortality rate for lymphoma and myeloma patients reported as 3.83 per 100,000 in 2017 [1]. The detection of minimal residual disease (MRD) plays an invaluable role in the comprehensive clinical management of these diseases, encompassing prevention, initial diagnosis, follow‐up treatment, prognosis, and recurrence monitoring. Effective MRD detection facilitates improved treatment outcomes, long‐term survival, and even potential clinical cures.

MRD refers to the small number of cancer cells that persist after initial treatment in patients who have achieved clinical and hematological remission, particularly in acute leukemia [2, 3, 4]. These residual cells are like small, undetectable specks of dust that remain even after a thorough cleaning‐barely visible but potentially significant. They represent a latent reservoir of disease that can lead to relapse if not properly addressed.

Accurate and early detection of MRD is crucial because it allows clinicians to identify and address these residual cancer cells before they grow into a full‐blown relapse. While MRD originally emerged as a concept in the context of hematological malignancies, it is also relevant in the management of various solid tumors, both benign (e.g., leiomyoma) and malignant (e.g., lung cancer, colon cancer, and ovarian cancer). The ability to detect MRD, even in the absence of symptoms, provides critical information that traditional methods may miss. This capability is crucial for assessing the effectiveness of treatment, predicting relapse, and guiding clinical trial endpoints for cancer drugs.

**FIGURE 1:**
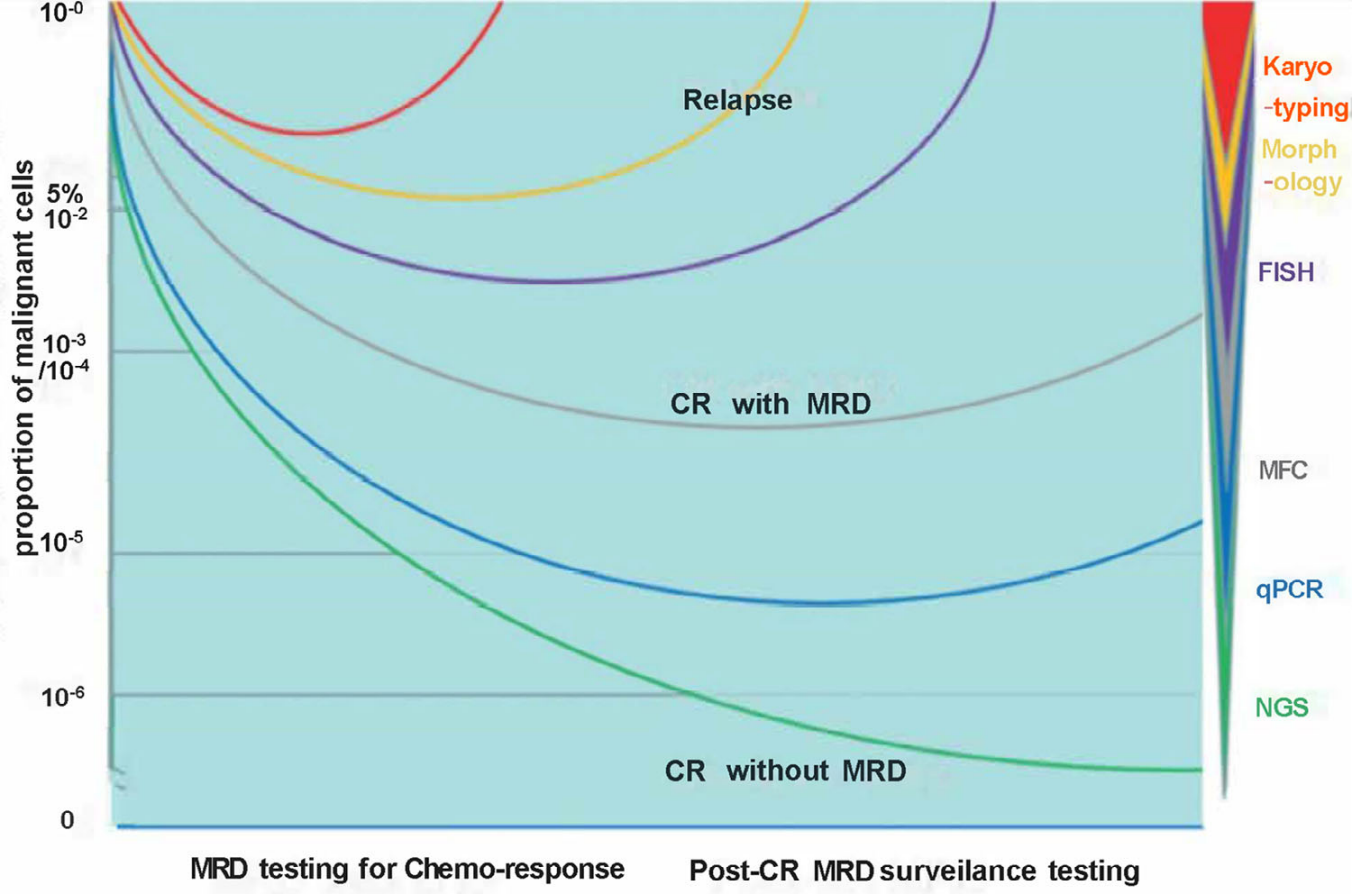
Results of treatment response testing and relapse patterns in patients with minimal residual disease (MRD) using different techniques with varying sensitivity levels. MRD detection is critical for monitoring treatment response and predicting relapse risk. A deeper response correlates with a better prognosis. However, even patients who achieve complete remission (CR) may relapse, suggesting that residual cancer cells may still be present. The level of MRD is closely associated with relapse risk. *Abbreviations*: complete remission (CR); fluorescence in situ hybridization (FISH); multiparameter flow cytometry (MFC); quantitative polymerases chain reaction (qPCR); next‐generation sequencing (NGS); minimal residual disease (MRD).

Early detection and intervention can dramatically improve patient outcomes by tailoring treatment strategies to the individual's current disease state, ultimately aiming to prevent recurrence and enhance overall survival (OS). Consequently, MRD detection results play a pivotal role in evaluating clinical trial endpoints for cancer drugs and determining the prognosis of cancer patients [5]. While many patients initially achieve remission through targeted treatments, the risk of relapse remains if signs of drug‐resistant disease eradication are absent. To effectively assess treatment outcomes and predict relapse, detecting malignant cells remaining in the body is essential (see Figure 1) [5, 6, 7, 8, 9, 10, 11, 12]. Monitoring MRD status at different stages of treatment and remission aids in understanding the disease status in hematological malignancies like acute or chronic lymphoblastic leukemia, acute or chronic myeloid leukemia, and MM.

Continuous monitoring of MRD status during and after treatment emerges as a key prognostic factor. It not only helps predict disease recurrence and assess therapeutic efficacy but also identifies patients at high and low recurrence risk, guiding treatment adjustments and offering insights for risk stratification [13]. Individualized studies in acute myeloid leukemia (AML) demonstrate a close relationship between detected MRD levels and OS and progression‐free survival (PFS). For instance, Berry et al. [14] found higher diseases incidence and lower survival rates in MRD‐positive children compared with MRD‐negative counterparts.

Clinical treatment of hematological tumors often lacks consideration for residual leukemia cell quantities, leading to insufficient treatment in patients with high MRD levels and unnecessary toxicity in those with low or no MRD. Hence, MRD detection becomes imperative, enhancing diagnostic and treatment accuracy and facilitating individualized precision treatment. This, in turn, significantly improves clinical efficacy, bearing great significance for disease treatment advancements [5].

This review outlines the principles, advantages, limitations, and applications of common MRD tests. Special attention is given to the quantitative detection, sensitivity, and clinical applicability of MRD, exploring the delicate balance generality and specificity in these methods. Given the profound impact of hematological tumor diseases, this review discusses the principles of current common MRD detection methods, expounds on their clinical application value, and analyzes the challenges and future directions of MRD detection.

## 2 Current MRD Detection Methods

MRD detection is a crucial aspect of diagnosing, monitoring, and treating hematological malignancies. Various techniques are employed, each offering distinct advantages and limitations. Karyotype analysis is traditionally used for diagnosing major chromosomal abnormalities but has limited sensitivity for MRD detection due to its inability to identify low levels of residual disease [15]. Fluorescence in situ hybridization (FISH) is effective for detecting specific genetic abnormalities and chromosomal translocations but typically has a sensitivity of around 10^−4^, which may not be sufficient for detecting MRD [16]. Flow cytometry (FCM), widely used for MRD detection, also offers sensitivity up to 10^−4^. It can identify abnormal cell populations, but its effectiveness can be affected by variations in antibody panels and gating strategies, and it may not detect all disease subtypes [17].

Quantitative real‐time polymerase chain reaction (qPCR) is an advanced form of PCR used to quantify the amount of a specific deoxyribonucleic acid (DNA) or ribonucleic acid (RNA) target present in a sample, providing quantitative data that reflects the initial amount of target nucleic acid in the sample. qPCR methods, such as fusion gene qPCR and immunoglobulin heavy chain (IgH)/T‐cell receptor (TCR) rearrangement qPCR, provide higher sensitivity for detecting specific genetic abnormalities. Fusion gene qPCR, used for targeting B‐cell receptor Ableson murine leukemia viral oncogene homolog 1, or BCR–ABL1, achieves sensitivity up to 10^−6^ and is valuable for monitoring known genetic abnormalities [18]. IgH/TCR rearrangement qPCR, which quantifies clonal rearrangements, also offers sensitivity up to 10^−5^ but may not detect all genetic variations [19].

Next‐generation sequencing (NGS), a modern DNA sequencing technology enables rapid and comprehensive analysis of genetic material. It can sequence millions of DNA fragments simultaneously. NGS stands out with its impressive sensitivity of up to 10^−6^. It allows for comprehensive detection of clonal rearrangements, somatic mutations, and MRD across a broad spectrum of genetic alterations. While NGS provides highly detailed insights into the clonal landscape of the disease, it is complex and requires sophisticated data analysis and interpretation [20].

The sensitivity and specificity of these MRD detection methods vary, and results may not always be consistent for the same patient at the same point. Therefore, selecting the appropriate detection method depends on the clinical scenario, including the type of malignancy and treatment context. Methods with higher sensitivity and specificity are generally more reliable for prognostic information, potentially leading to improved clinical outcomes through early intervention and personalized treatment [21, 22, 23]. Detailed comparisons of these techniques, including the applicability, sensitivity, advantages, and limitations, are summarized in Table 1 [8, 24, 25].

**TABLE 1:** Comparison of different MRD detection methods [8, 24, 25].

Platform	Applicability	Sensitivity	Advantages	Limitations
Karyotyping	∼50%	5 × 10^−2^	① Widely used ② Standardized	① Slow report time ② High demand for labor ③ Requires preexisting abnormal karyotype
FISH	∼50%	10^−2^	① Useful for quantifying cytogenetic abnormalities ② Relatively fast report time	① High demand for labor ② Requires preexisting abnormal karyotype
RT‐qPCR	∼40–50%	10^−4^–10^−6^	① Widely used ② Standardized ③ Lower costs	① Only one gene assessed per assay ② Mutations outside the region spanned by the gene primer are easily overlooked
FCM	Almost 100%	3–4 colors: 10^−3^–10^−4^ 6–8 colors: 10^−4^ ≥8 colors: 10^−4^–10^−6^	① Widely used ② Relatively fast report time ③ Wide range of application ④ Relatively inexpensive	① Lack of standardization ② Required professional knowledge ③ Changes in immunophenotype ④ Fresh cells required
NGS	>95%	10^−2^–10^−6^	① Multiple genes analyzed at once ② Can detect mutations in the tested part of the gene ③ Broad applicability	① Not widely used ② Slow report time ③ Not standardized yet ④ High cost ⑤ Required professional knowledge ⑥ Requires diagnostic pretreatment sample

Sensitivity is defined as the ability of a method to detect 1 leukemic cell in a maximum of X cells.

*Abbreviations*: Minimal residual disease (MRD); fluorescence in situ hybridization (FISH); real‐time quantitative reverse transcription polymerases chain reaction (RT‐qPCR); flow cytometry (FCM); next‐generation sequencing (NGS).

### 2.1 Traditional Morphological Method

Bone marrow cytologic examination has long been the gold standard for assessing complete remission (CR) in the treatment of childhood leukemia. In clinical studies, CR is typically defined as having less than or equal to 5% leukemic blasts in the bone marrow [26]. However, this approach has notable limitations due to its relatively low sensitivity. The actual burden of leukemia cells in the body can vary widely, ranging from negligible levels to as high as 10^9^ cells, depending on individual treatment responses and disease progression. This variability means that traditional morphology‐based methods may fail to detect MRD accurately, leading to potential misjudgments in treatment planning. For example, elevated MRD levels may indicate insufficient chemotherapy, increasing the risk of relapse, while undetected residual disease could lead to overtreatment, resulting in severe side effects and complications that could affect long‐term survival. Thus, while traditional morphological methods are useful for initial remission assessment, they may not be sufficiently sensitive to guide ongoing treatment decisions and ensure optimal patient outcomes.

### 2.2 Cytogenetic Method

Karyotype analysis is a classical method used to identify chromosomal aberrations in the bone marrow and peripheral blood cells of leukemia patients. This technique focuses on detecting abnormal chromosomal structures during both the diagnostic phase and throughout treatment. Patients exhibiting early chromosomal abnormalities often show a correlation between achieving morphological CR and the disappearance of these abnormal karyotypes. Despite its diagnostic value, this method has significant limitations. Karyotype analysis is dependent on the proliferation rate of the detected cells and can only assess cells in metaphase, restricting its utility to a specific phase of cell division. Furthermore, its sensitivity is limited, ranging between 10^−2^ and 10^−1^, meaning it may miss low levels of residual disease. Although it can offer insights into the potential for short‐term relapse in patients who have achieved CR, the low sensitivity and phase‐specific limitations reduce its clinical applicability, particularly in cases where more sensitive MRD detection is needed.

### 2.3 Fluorescence in Situ Hybridization

**FIGURE 2:**
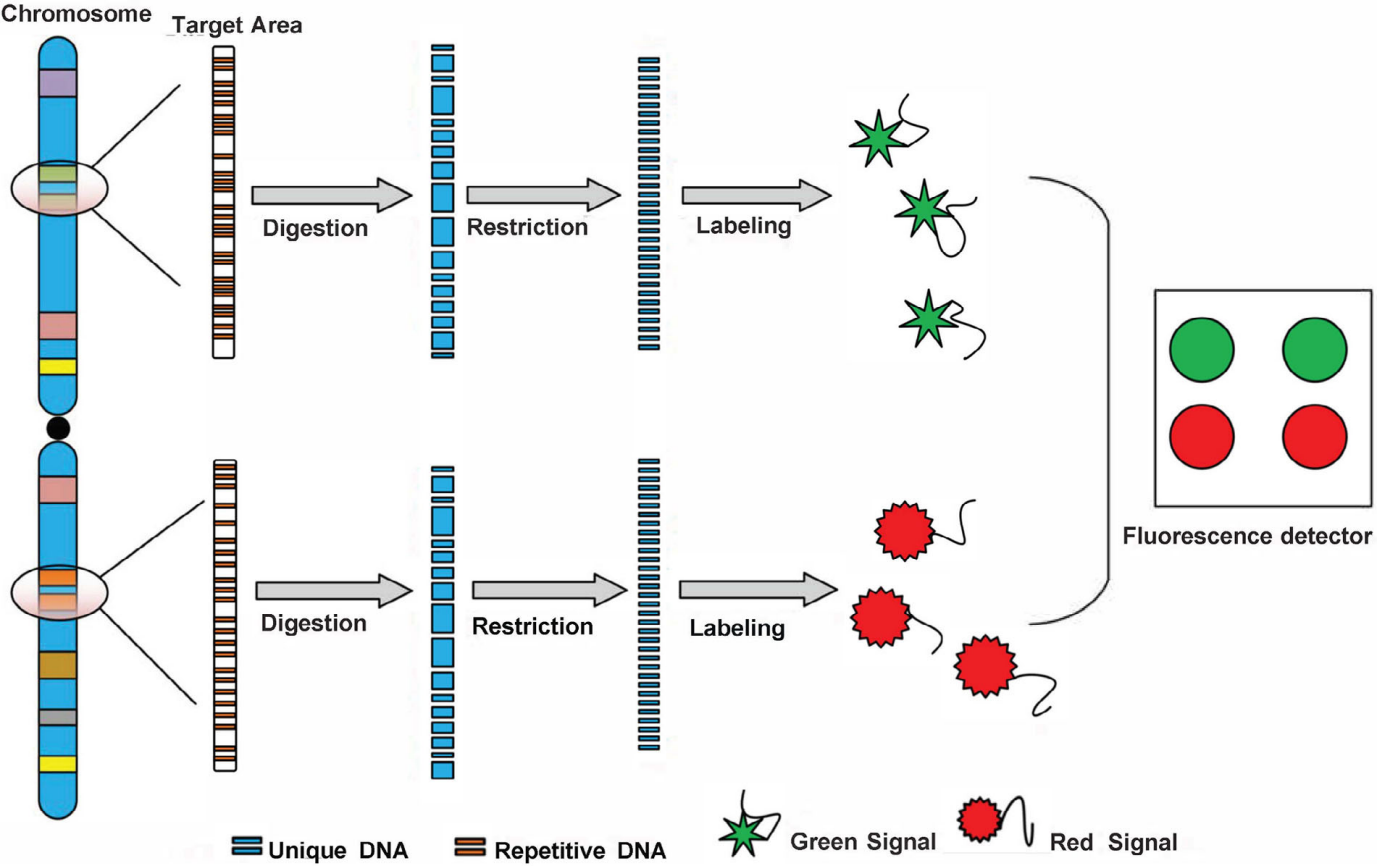
Technical principle of fluorescence in situ hybridization (FISH). FISH uses fluorescently labeled DNA probes to hybridize with specific DNA or RNA sequences in the sample, allowing for the detection and localization of genetic material within cells.

Developed in the late 1980s, FISH emerged as a nonradioactive molecular biology and genetics technique that uses fluorescently labeled nucleic acid probes to bind directly or indirectly to specific DNA target sequences within the cell nucleus. Based on the principle of complementary base pairing, FISH enables qualitative, quantitative, and relative localization of chromosomal or genetic abnormalities within cells. This method addresses some of the limitations of traditional cytogenetic techniques and provides valuable information regarding chromosomal and gene status within the cell nucleus (see Figure 2) [27].

In MRD detection, FISH commonly uses single‐sequence probes designed to target chromosomal translocation breakpoints, such as BCR = ABL and PML–RARA fusion genes, which are important markers in leukemia. Unlike traditional cytogenetic methods, FISH can capture information from interphase cells, increasing the ability to detect chromosomal numerical or structural changes even in low‐proliferation cell [28]. This method proves suitable for tracking MRD in patients after hematopoietic stem cell transplants.

However, FISH has several limitations. Its sensitivity, ranging from 10^−2^ to 10^−3^, can be impacted by the presence of nonleukemic aneuploid cells and technical challenges during the detection process. While polychromatic FISH improves sensitivity, it remains prohibitively expensive due to the cost of labeled probes, making it less practical for routine MRD detection in acute leukemia patients.

Studies in cell biology over the past two decades have shown that when the total number of leukemia cells in the body is less than 10^6^ (weighing under 1 mg), the body's immune system may be able to eliminate the residual leukemia cells. Given this, the sensitivity levels provided by FISH and similar methods are inadequate to meet clinical needs. More sensitive techniques from molecular immunology and molecular biology, or a fusion of both, are required to accurately detect MRD in acute leukemia cases.

### 2.4 Flow Cytometry

#### 2.4.1 Detection Principle of FCM in MRD Assessment

FCM is an advanced technology that enables the rapid measurement of various biological properties at the single‐cell level, facilitating the quantification and sorting of cells without damaging their structure. In the context of MRD detection, FCM operates by identifying abnormal immunophenotypes that are characteristic of leukemia cells. This is achieved by analyzing the expression patterns of a series of antigens on the cell surface or intracellular surface, allowing for a detailed multiparameter quantitative analysis known as “leukemia‐associated immunophenotypes” (LAIPs) [2, 29].

#### 2.4.2 Antibody Combination Selection for MRD Detection

FCM enables the semi‐quantification of protein expression on the cell surface or within the cytoplasm by measuring the binding of fluorescently labeled antibodies [30]. In MRD detection, the selection of antibody combinations is critical and is guided by specific principles depending on the method used for detecting MRD.

##### 2.4.2.1 Reference to Initial LAIP

When the initial LAIP is available for reference, the antigens involved in that immunophenotype should be included in the MRD detection antibody combination. This allows for accurate tracking of residual leukemia cells based on the patient's initial disease profile.

##### 2.4.2.2 Patients Without Initial Immunophenotype

In cases where the initial immunophenotype is unavailable, reference can be made to the frequency of LAIPs in the same type of leukemia after treatment. In these instances, selecting antigens with high frequency and including a broad panel of antibodies reduces the risk of missed detection of residual disease.

There are two main approaches to analyzing MRD by FCM: tracking cells with the known LAIP and identifying differences from normal (DFN) clones. The DFN strategy is especially important because individual cell phenotypes may change after induction therapy, requiring more than just tracking the original LAIP. Sometimes, DFN is used in combination with the LAIP approach for enhanced detection.

Key reagents for FCM studies include fluorochrome‐conjugated antibodies that target cell surface, cytoplasmic, or nuclear antigens. Table 2 outlines the antigens commonly evaluated in hematologic malignancies [31]. The selection of the antibody panel should be disease‐specific and adjusted based on the patient's phenotypic characteristics at diagnosis [32].

**TABLE 2:** Surface or intracellular antigens commonly assessed by flow cytometry for lineage classification and immunophenotyping of hematolymphoid malignant neoplasms [31].

Lineage	Antigens
Stem cells	CD34, CD38, CD45
B cells	CD5, CD10, **CD19, CD20, CD22**, CD23, CD25, CD34, CD38, CD43, CD45, **CD79a**, CD103, CD200, FMC2, **cIgM, Kappa, Lambda**, LEF1, TdT
Plasma cells	CD19, CD20, CD38, CD45, CD56, CD117, CD138, **cKappa, cLambda**
T cells/NK cells	CD1a, **CD2, CD3**, CD4, **CD5**, CD7, **CD8**, CD10, CD16, CD25, CD26, CD30, CD34, CD45, CD56, **αβ‐TCR, γδ‐TCR**, TdT, **TRBC1**
Myelomonocytic cells	CD4, CD7, CD10, CD11b, CD11c, **CD13, CD14**, CD15, CD16, **CD33**, CD34, CD36, CD38, CD45, CD56, **CD64, CD65**, CD71, CD117, CD123, **cMPO, cLyso**, HLA‐DR
Erythroblasts	CD34, CD36, CD38, CD45, CD71, CD117, **CD235a**
Megakaryoblasts	CD33, CD34, CD38, **CD41, CD42**, CD45**, CD61**, CD117, HLA‐DR

The antigens in bold are lineage‐specific markers.

*Abbreviations*: cytoplasmic IgM (cIgM); cytoplasmic kappa (cKappa); cytoplasmic lambda (cLambda); cytoplasmic lysozyme (cLyso); cytoplasmic peroxidase (cMPO); T‐cell receptor (TCR); terminal deoxynucleotidyl transferase (TdT); TCR β chain constant 1 (TRBC1).

#### 2.4.3 Advantages and Limitations of FCM

FCM offers significant advantages as a highly sensitive technique, enabling rapid, multiparameter analysis and sorting of small particles such as cells or microorganisms. This technology is frequently employed for rapid immunophenotypic analysis, and boasts the capability to quickly determine crucial parameters, segregate distinct cell properties without causing cellular damage, and ultimately yield pure cell populations for biological and medical research. With a current maximum sorting speed of up to 30,000 cells per second, FCM is an invaluable tool in both biological and medical research. Its advantages include prompt detection, wide applicability across various fields, and cost effectiveness, making it a popular choice in clinical and laboratory settings [33]. With continuous advancements, the second generation of FCM is becoming increasingly integrated into routine clinical applications [34].

However, despite these strengths, FCM has significant limitations. One major challenge is that the cellular immunophenotype of cells at diagnosis may differ from those in relapsed disease, especially posttreatment with monoclonal antibodies [35]. This can complicate MRD detection and reduce accuracy. Additionally, the sensitivity of FCM is contingent on several factors, including the number of viable cells obtained, the degree of tumor cell abnormality, and the proportion of normal B cell precursors present in the subsequent experimental sample. Achieving a sensitivity of 10^−4^ requires analyzing at least 100,000 cells per sample [36, 37]. This demand can be burdensome for patients, as large sample sizes are needed, and samples must be processed quickly to maintain cell viability (which typically requires at least 85% survival) [38].

Last, a major limitation of FCM is the lack of standardization across MRD tests. Significant variation exists in the choice of markers and antibody panels, the number of cells tested, and the criteria used to define MRD positivity. Three inconsistencies can affect the comparability of results between different laboratories and studies, limiting the widespread adoption of FCM as a uniform standard for MRD detection [39].

#### 2.4.4 Factors Influencing False‐Negative MRD Detection in FCM

False‐negative results in MRD detection via FCM are influenced by several critical factors.

##### 2.4.4.1 Sensitivity

The sensitivity of FCM typically ranges between 10^−4^ and 10^−5^. While strategies involving multiparameter and multigate analysis can improve sensitivity, it is not infinite. Leukemia cells that exist below this sensitivity threshold may go undetected, leading to false negative results. This limitation is particularly concerning in cases where low levels of residual disease persist after treatment.

##### 2.4.4.2 High Heterogeneity of Leukemia

Leukemia is a heterogenous disease, with some patients displaying no abnormal immunophenotype or presenting varying subtypes. During treatment, immunophenotypic shifts may occur, resulting in a loss of white blood cell phenotypic specificity within disease cell clones. These transformations make it difficult to detect residual malignant cells and increase the likelihood of false negatives. To mitigate this risk, research centers often recommend assessing two different immunophenotypes for each patient, which helps improve the reliability of the results.

##### 2.4.4.3 Conventional MRD Analyses

Traditional MRD detection requires multiple invasive bone marrow aspirations. However, bone marrow diseases are known to exhibit multifocal patterns, with disease cells often forming “plaques” in different areas of the marrow. This uneven distribution of leukemia cells can lead to sampling errors, where certain bone marrow regions may appear free of disease, despite the presence of residual cancer cells elsewhere. Consequently, these multifocal characteristics contribute to false‐negative MRD results [40].

These challenges highlight the need for enhanced methodologies or complementary approaches to ensure accurate and reliable MRD detection.

### 2.5 Polymerase Chain Reaction

PCR is a widely used molecular biology technique for amplifying specific DNA sequences, it exponentially replicates a targeted segment of DNA allowing for generation of millions of copies of that segment from a small initial sample. PCR has played a pivotal role in both past and current MRD detection scientific research, offering high sensitivity and specificity in identifying residual disease [27, 41–44]. In MRD detection, PCR is used to amplify and detect tumor‐specific sequences, including antigen receptor gene rearrangements, chromosomal translocations, and fusion genes associated with leukemia cells. This approach is highly valued for its simplicity, speed, and ability to achieve sensitivity levels between 10^−4^ and 10^−6^, making it one of the most reliable techniques for detecting low levels of residual disease in hematological malignancies. PCR‐based MRD assays have proven especially effective in the majority of acute lymphoblastic leukemia (ALL) patients, though their application in AML remains more challenging due to the heterogeneity of genetic markers.

Several PCR‐based are employed for MRD detection, including nested PCR, fluorescent quantitative PCR, and semi‐quantitative PCR. Among these, real‐time quantitative PCR (RQ‐PCR) is considered the most sensitive, boasting a detection threshold as low as 0.001–0.0001%. RQ‐PCR detects MRD through the amplification of fusion genes formed by immunoglobulin (Ig) or T cell receptor (TCR) gene rearrangements, as well as chromosomal translocations specific to leukemia. The quantitative nature of RQ‐PCR allows for precise measurement of MRD levels, facilitating a better understanding of treatment response and relapse risk. Additionally, the ability to quantify residual disease enables more personalized treatment strategies and risk stratification, making PCR a crucial tool in the ongoing management of leukemia patients.

#### 2.5.1 Ig/TCR Rearrangement in MRD Detection

Antigen receptors on the surface of lymphocytes, such as the TCR and BCR, are crucial surface markers whose formation is controlled by genes in the nucleus. The encoding of the TCR and BCR involves multiple gene rearrangements during lymphocyte development. This process is highly specific and involves the recombination of variable (V), diversity (D), and joining (J) gene segments to form unique gene sequences. These rearrangements, combined with random base loss or insertion at the junction (N‐region), as well as somatic mutations, create extensive receptor diversity. As a result, the unique Ig/TCR rearrangements serve as highly specific markers for individual B or T cell clones, making them reliable tumor‐specific targets for detecting MRD.

In ALL and some cases of AML, TCR gene rearrangements are commonly present, making them key targets for PCR‐based MRD detection. Ig or TCR gene sequences are patient specific, and their persistence after treatment can indicate the presence of residual malignant cells. PCR detection of these rearrangements is a widely adopted method for MRD monitoring in ALL patients, leveraging the high specificity of the Ig/TCR gene as a marker for residual leukemic cells.

Recent research reveals that Ig/TCR gene rearrangement in lymphoid tumors can result in the formation of subclones. These subclones may undergo secondary rearrangements, also known as oligoclonality. This clonal evolution is often observed during disease progression, with new clones emerging as the disease advances. Oligoclonality and clonal evolution present a challenge for MRD detection, as they can lead to false‐negative results if newly formed subclones are not detected postchemotherapy. To mitigate this, it is recommended to simultaneous target at least two Ig/TCR gene rearrangements in MRD assays to reduce the likelihood of false negatives and improve the sensitivity of MRD detection [45].

#### 2.5.2 Chromosomal Translocations and Corresponding Fusion Genes

Chromosome translocation, which involve the rearrangement of chromosomal segments, often leads to abnormal gene structure or altered gene expression at or near the fracture point. These alterations are closely associated with leukemia progression and serve as critical targets for MRD detection. Primers designed based on gene sequences near the two chromosome break sites are utilized for RQ‐PCR to amplify the transcripts of leukemia‐specific fusion genes, offering a highly sensitive method for MRD monitoring.

A well‐known example of this is the BCR–ABL fusion gene, formed by the t (9;22) translocation, commonly known as the Philadelphia chromosome (Ph). Although present in only 3–5% of childhood ALL cases, the BCR–ABL fusion gene is a critical target for quantitative MRD detection due to its strong association with poor prognosis. Similarly, the TEL–AML1 fusion gene, resulting from the t (12;21) translocation, is present in approximately 23% of children with pre‐B‐ALL and serves as a marker for MRD monitoring in this subgroup. Another example is the t (4;11) (q21; q23) translocation, which generates the MLL–AF4 fusion gene, prevalent in 70–80% of infants with ALL, where it is associated with a high risk of relapse and poor prognosis. For AML patients carrying the MLL–AF4 translocation, this fusion gene also serves as an ideal target gene for MRD detection [46].

Fusion genes offer a unique advantage in MRD detection because they directly reflect the genetic changes driving the leukemia, making the assay both highly specific and sensitive, with detection limits ranging from 10^−4^ to 10^−6^. However, a limitation of this approach is that it only applies to leukemia patients who harbor specific chromosomal translocations. For those without fusion genes or with translocations where the breakpoints are unclear, this method is less useful for MRD monitoring. Consequently, while fusion gene‐based PCR is a powerful tool, its utility is confined to a subset of leukemia cases.

#### 2.5.3 Challenges and Solutions of PCR

##### 2.5.3.1| False Negatives

One of the main challenges in MRD detection using Ig and TCR gene rearrangements is the occurrence of clonal evolution during disease progression. As the leukemia evolves, new subclones may emerge, leading to changes in the Ig or TCR gene rearrangement patters, potentially rendering previously identified monoclonal markers ineffective. This clonal evolution can result in false‐negative MRD tests, where residual disease is present but undetected due to these changes in the leukemic clone's genetic makeup. To mitigate the risk of false negatives, it is recommended to employ two or more independent monoclonal Ig/TCR markers for MRD detection assays. This redundancy increases the likelihood of capturing evolving clones and ensures a more reliable assessment of residual disease.

##### 2.5.3.1 Contamination Risks

PCR‐based detection of fusion gene transcripts, such as those formed by chromosomal translocations, offers sensitivity level comparable to Ig/TCR‐based targets. However, fusion gene transcripts degrade rapidly in vitro, which can compromise the accuracy of MRD detection. Furthermore, fusion transcript detection rates hover around 50%, increasing the risk of contamination. Unlike Ig/TCR markers, fusion genes are not patient specific, which makes contamination more likely when dealing with samples from multiple patients. To reduce the risk of contamination, it is crucial to process samples as soon as possible after collection. Ideally the samples should be analyzed on the same day to prevent transcript degradation and minimize handling errors that could introduce contaminants [47, 48, 49].

Incorporating strict contamination controls, including the use of separate workspaces and reagents for pre‐ and post‐PCR processes, can also help mitigate contamination risks. Adhering to these strategies ensures that MRD detection methods retain their high sensitivity and specificity while minimizing false negatives and contamination.

### 2.6 NGS and Emerging Techniques

NGS has revolutionized the field of molecular diagnostics, offering a high‐throughput analysis method capable of simultaneously reading and analyzing numerous DNA fragments with unparalleled precision [43, 50]. NGS provides accurate and detailed DNA sequence information, detecting relevant variants like insertions, deletions, exons mutations, gene rearrangements, and large‐scale genome losses. The method's capacity to achieve deep sequencing coverage (where coverage refers to the number of times a base sequence is read during sequencing) directly correlates with the accuracy of the resulting data [51]. Higher coverage enhances confidence in detecting rate or low‐frequency variants, which is crucial for identifying MRD. NGS proves valuable for assessing replication changes, epigenetic changes, and various mutations in leukemia patients’ cell populations between cured and relapsed cells [22, 52–55, 56, 57].

NGS has become a powerful tool in MRD detection due to its sensitivity, which can reach levels as low as 10^−6^, detecting a single leukemic cell in a background of one million healthy cells. This makes it particularly valuable for monitoring residual disease in hematological malignancies, especially in cases where traditional methods fall short. NGS can assess gene rearrangements in both BCR and TCR genes, enabling the identification of clonal populations within a patients’ immune repertoire. This specificity is advantageous for tracking clonal evolution, relapse, and resistance mechanisms during and after treatment.

Emerging NGS techniques have also begun to integrate unique molecular identifiers (UMIs) into sequencing workflows, increasing the precision of MRD detection by reducing amplification and sequencing errors. iRepertoire Inc. has introduced a novel BCR–IgH panel that specifically targets the V(D)J regions of gDNA using their proprietary dimer‐avoided multiplex PCR technology [58]. The addition of UMIs ensures that each V(D)J sequence is linked to a single cell, preserving the one V(D)J per cell relationship. This allows for accurate cell counting and eliminates PCR and sequencing errors, resulting in more precise analysis of low‐frequency clones and hypermutations within the BCR–IgH repertoire.

This method represents a significant leap in MRD detection, providing not only a detailed view of the BCR repertoire but also enabling the true cell frequency to be captured, a first for such assays. This advancement holds the potential to improve the sensitivity and accuracy of MRD detection in clinical settings, especially for cases involving low tumor burden or hypermutated clones. As this technology matures, it may pave the way for its broader application beyond hematological malignancies, enhancing our understanding of immune diversity and disease relapse mechanisms.

#### 2.6.1 Basic Principle and Detection Significance of NGS

**FIGURE 3:**
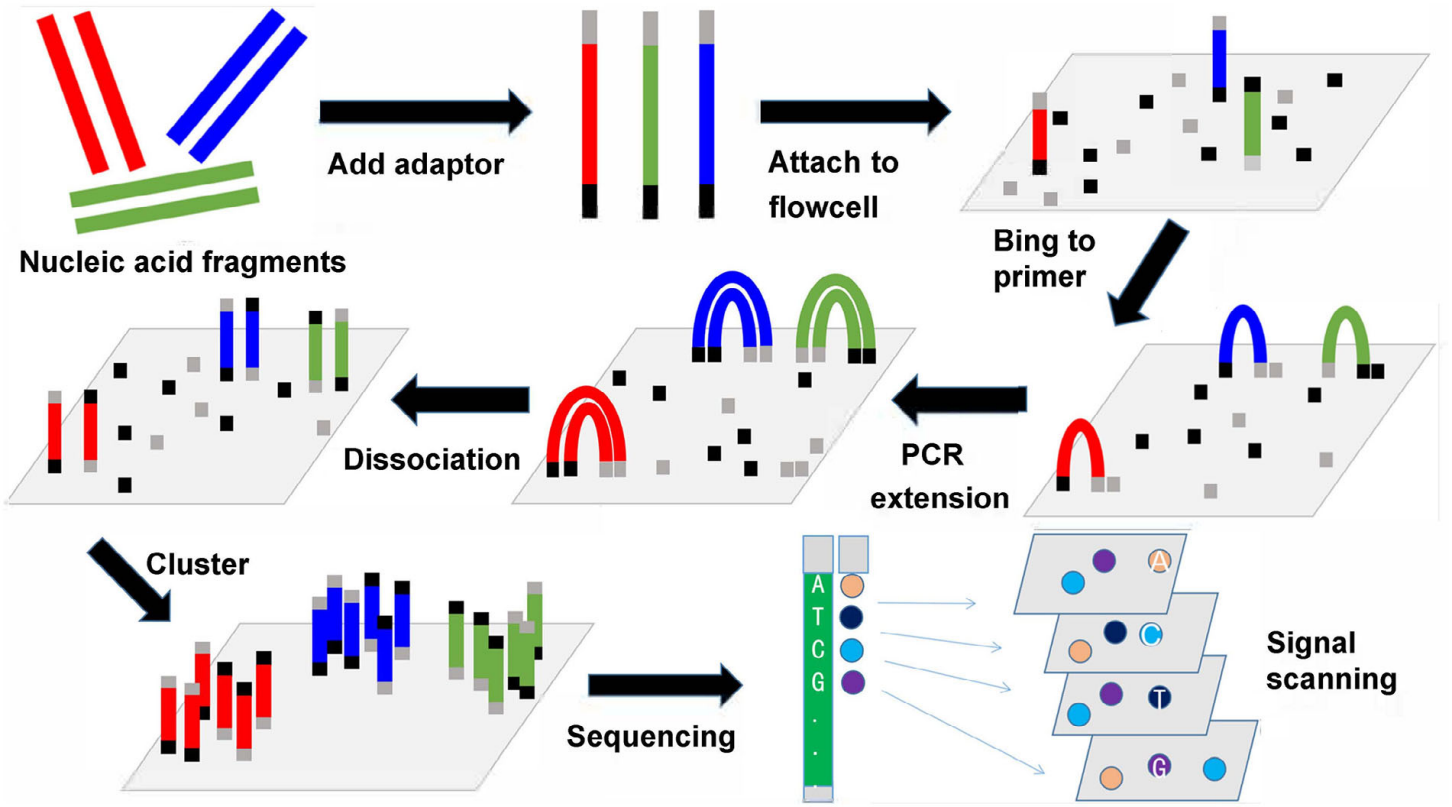
Workflow of next‐generation sequencing (NGS) process. NGS involves adding adapters to fragmented nucleic acids, amplifying them with complementary primers, enriching the library, performing sequencing, and analyzing the results through signal scanning.

The NGS process involves modifying DNA molecules and immobilizing them on nanopores or microcarriers chips (Figure 3) [27]. The sequencing process relies on the principle of base complementarity, where the accurate pairing of nucleotide bases (A‐T, C‐G) is tracked through the detection of fluorescence or chemical reactions during PCR amplification or ligase‐mediated reactions. This process enables the precise reading of DNA sequences and provides a comprehensive analysis of genetic information.

In MRD detection, NGS is applied to sequence a vast number of rearranged V‐(D)‐J fragments from the Ig and TCR genes. By sequencing these gene fragments, NGS not only detects residual leukemia cells but also provides a window into the immune repertoire of the patient's cells [59]. This method is particularly useful in identifying specific clonal populations that remain after treatment, allowing for a detailed assessment of disease progression or remission.

The ability of NGS to monitor MRD levels has significant prognostic value. NGS‐based MRD monitoring offers improved sensitivity over traditional methods, providing a more reliable indicator of early relapse risk. Furthermore, NGS can redefine MRD negativity, particularly in cases where patients were previously classified as MRD‐positive by other molecular methods, reducing the occurrence of false positives [60]. This enhanced sensitivity and accuracy in MRD detection have made NGS an indispensable tool for guiding personalized treatment decisions, improving patient outcomes, and refining risk stratification in hematological malignancies.

#### 2.6.2 Advantages of NGS in MRD Detection

NGS offers several advantages for MRD detection, particularly in its high sensitivity and precision. Using a common set of primers, enabling NGS can accurately identify unique tumor‐specific targets, enabling it to detect MRD with an exceptional sensitivity ranging from 10^−4^ to 10^−6^ [61]. One study, which analyzed 236 bone marrow samples from 64 patients with ALL, demonstrated that after chemotherapy, NGS detected MRD in 57.5% of B‐ALL positive cases and 80% of T‐ALL positive cases‐ significantly higher detection rates compared with multiparameter flow cytometry (MFC) and RQ‐PCR. This clearly illustrates the heightened sensitivity of NGS in MRD detection [23].

In another study by Huang et al. [62], MRD detection on 63 samples revealed that patients who tested positive by NGS but negative by MFC had lower leukemia‐free survival rates (*p* = 0.037), suggesting that NGS is more effective in monitoring tumor burden and providing valuable prognostic insights. Similarly, a comparison between NGS and FCM for MRD detection in relapsed patients showed that the detection rates for MRD using FCM, NGS with a sensitivity of 10^−4^ and NGS with a sensitivity of 10^−6^ were 50, 69, and 100%, respectively, highlighting NGS's superior ability to predict the risk of relapse [53].

Additionally, NGS sensitivity allows for the quantification of MRD in peripheral blood at levels more than ten times lower than those detectable in bone marrow, expanding its utility in clinical settings [63, 64, 65, 66, 67]. Another key advantage is its ability to detect rearranged clonal link‐region sequences of Ig/TCR genes in almost all leukemia patients, further enhancing the clinical applicability of Ig/TCR‐based MRD detection [68].

#### 2.6.3 Challenges and Solutions

##### 2.6.3.1 Threshold Value

One of the ongoing debates in NGS‐based MRD detection is the appropriate threshold for identifying Ig/TCR clones in ALL patients. Current methods often set thresholds at 5% [52, 69, 70] or 10% [71] as for clonal identification, but the optimal threshold remains unclear, if it should vary based on the leukemia's extent in clinical samples or whether it should remain the same. Determining an optimal threshold is crucial for improving diagnostic accuracy and remains a topic of research and discussion.

##### 2.6.3.2 Sensitivity and Disease Burden Quantification

**FIGURE 4:**
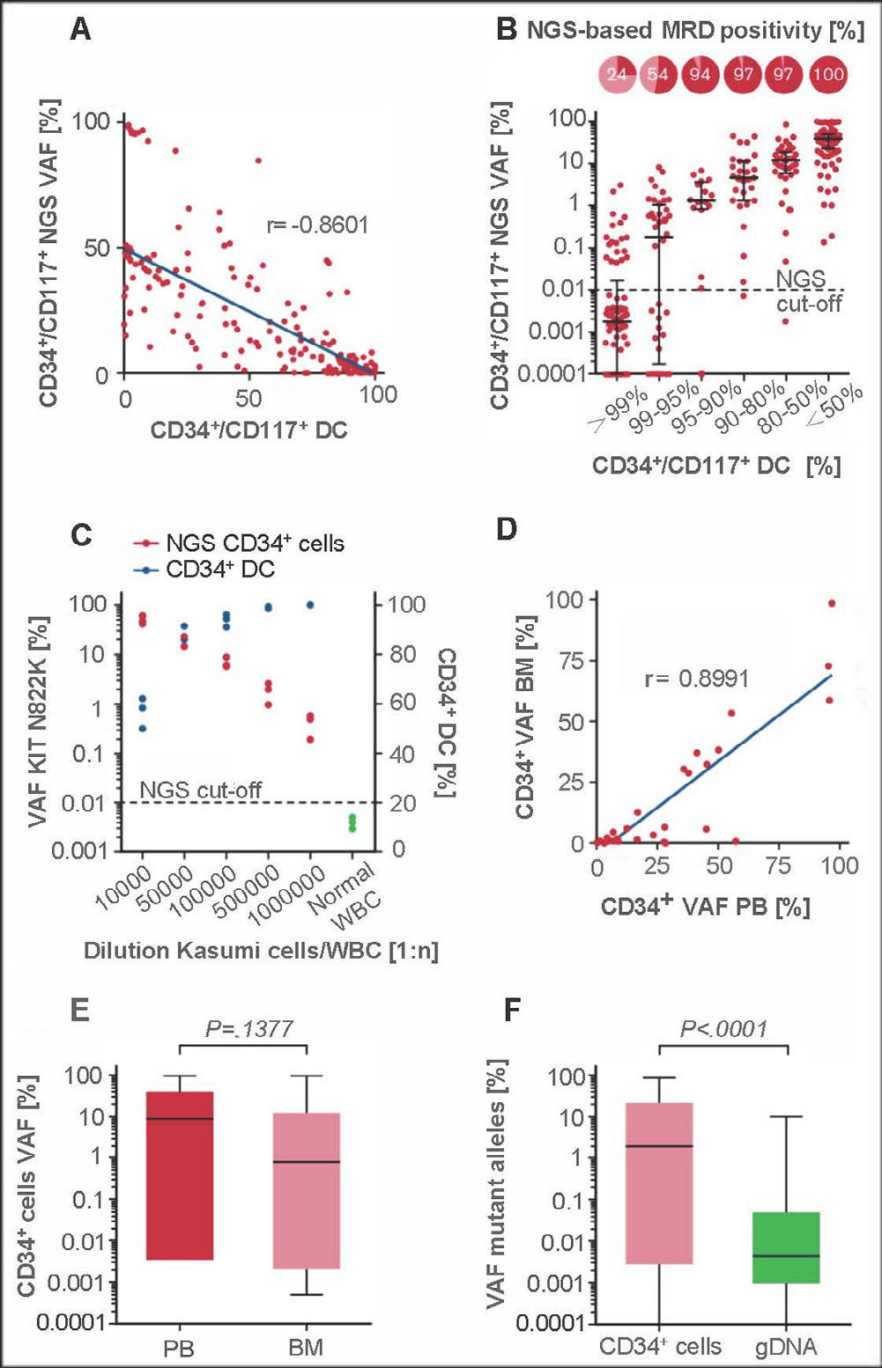
Evaluation of NGS‐based MRD detection in CD34^+^/CD117^+^ cells. This figure compares NGS‐based MRD detection with donor chimerism (DC) analysis, highlighting sensitivity in peripheral blood (PB) and bone marrow (BM) samples. NGS‐based detection shows high correlation with DC analysis in sorted CD34^+^/CD117^+^ PB cells, with better sensitivity when initial FACS enrichment was performed. (A) Correlation of MRD detection using NGS or DC analysis in sorted CD34^+^/CD117^+^ PB cell samples. (B) NGS‐based MRD positivity rates in relation to the corresponding CD34^+^/CD117^+^ DC level in PB. The cutoff for NGS‐based MRD quantification is indicated at 0.01% VAF. (C) Detection of the Kasumi cell line in PB using NGS‐based quantification of the KIT N822K variant (red dots) or by CD34^+^ DC analysis (blue dots). (D) Correlation of NGS‐based MRD detection in sorted CD34^+^ cells of matched PB and BM samples as templates for analysis. (E) Quantification of variant allele frequencies (%) in CD34^+^ cells using matched PB or BM samples for NGS. (F) Quantification of mutant alleles by NGS using sorted CD34^+^/CD117^+^ PB cells or unsorted material of matched follow‐up samples. Box plots represent median values with interquartile range; box whiskers represent minimum to maximum values. *Abbreviations*: next‐generation sequencing (NGS); minimal residual disease (MRD); donor chimerism (DC); peripheral blood (PB); variant allele frequency (VAF). Reproduced with permission from Ref. [79], Copyright © 2022 The American Society of Hematology.

NGS achieves an exceptional sensitivity of up to 10^−7^, but this comes at the cost of requiring a significant amount of nucleic acid for analysis [52, 72, 73]. However, the large number of samples required makes the testing process cumbersome and expensive. Despite these challenges, NGS offers the benefit of quantifying MRD levels in peripheral blood, reducing the need for invasive bone marrow aspiration [74]. One study shows that MRD levels in peripheral blood and bone marrow are consistent over 2 years of treatment period in AML patients [75]. Another study comparing CD34^+^ donor chimerism and NGS showed that NGS has a lower detection limit, demonstrating between sensitivity for MRD detection in CD34^+^ cells from peripheral blood compared with bone marrow (Figure 4A,B,C). Combining the two tests to detect MRD in bone marrow and peripheral blood CD34^+^ cells, the results were strongly correlated, and MRD was more sensitive with the use of peripheral blood than with the use of bone marrow after initial fluorescence‐activated cell sorting (FACS) enrichment (Figure 4D,E,F) [76]. Sensitivity needs to reach the lowest limit when detecting MRD levels in bone marrow to accurately detect MRD while minimizing harm to patients. However, quantifying the exact copy number of target sequences remains a challenge due to issues like primer dimerization, nonspecific amplification, and amplification bias.

##### 2.6.3.3 Limitations on Specificity

The high sensitivity of NGS also presents challenges in distinguishing between true leukemia‐related clones and low abundance lymphoid clones that are unrelated to leukemia. Current studies usually exclude low‐cloning levels from analysis, instead relying on critical value to define the leukemia index sequences. For instance, Wu and colleagues found low‐level same cross‐patient clonality in TCR gene rearrangements (TRB and TRG) in 20 of 40 T‐ALL patients [77]. Defining an appropriate background frequency for index sequence could help mitigate this issue and improve specificity in NGS‐based MRD detection.

##### 2.6.3.4 Contamination Risks

NGS is highly sensitive, but this also makes it suspectable to contamination from amplified fragments and barcode misallocation between sequencing runs [78, 79, 80]. Illumina reports that, with proper cleaning and maintenance, the risk of run‐to‐run contamination is below 0.1%. To minimize cross‐contamination, different barcode combinations should be used between sequencing runs. Additionally, shared laboratory tools used in primer synthesis pose a contamination risk, so targeted cleaning protocols can help prevent oligonucleotide interactions [81]. Filtering barcode sequences with sufficient quality is another strategy to reduce barcode misallocation [82, 83].

In conclusion, while NGS holds great potential for MRD detection, addressing challenges such as standardization, amplification bias [84], sensitivity, specificity, contamination, and accurate quantification is critical for its successful clinical application [85].

## 3 Clinical Implications and Applications

More and more studies have shown that MRD response to first‐line therapy has become an independent predictor beyond traditional prognostic parameters [24], and has a wide range of clinical application prospects. MRD detection can not only be used as a prognostic/predictive biomarker to improve risk assessment and guide treatment decisions. In addition, MRD assessment can be used as a monitoring tool to identify impending relapse and as a potential surrogate endpoint for OS in clinical trials to accelerate the development of new therapeutic strategies [86, 87].

### 3.1 MRD as a Prognostic Biomarker

MFC has been widely utilized for MRD detection [88, 89, 90, 91]. It not only provides valuable prognostic information but also aids in guiding treatment decisions for patients with hematologic malignancies [92]. For instance, MFS has been instrumental in assessing MRD levels after induction therapy, which has been shown to correlate with patient outcomes. A significant study demonstrated that both MRD response and overall hematological response are independent prognostic factors [7, 93]. This finding underscores the importance of MRD detection in tailoring treatment strategies and highlights the need for standardization across different clinical settings to ensure consistent and reliable results.

In the AML17 trial, MRD was assessed by MFC in 2450 patients with high‐risk, wild‐type AML with NPM1 mutations. After one cycle of induction chemotherapy, patients who were MRD‐negative had significantly better OS rates (70 vs. 51% at 5 years; *p* < 0.001) compared with those with detectable MRD. After two treatment cycles, patients with MRD ≥1% had a higher risk of recurrence (89%) and a shorter OS than those with lower or undetectable MRD levels (33 vs. 63%, *p* = 0.003). Importantly, allogeneic hematopoietic stem cell transplant (allo‐HSCT) appeared to benefit patients with persistent MRD more than those who achieved MRD negativity, suggesting that allo‐HSCT should be prioritized for patients with standard‐risk, NPM1 wild‐type AML who remain MRD‐positive after induction therapy [94].

In MRD detection by PCR, the prognostic impact is particularly evident in studies of NPM1‐mutant AML. The NPM1 mutation is relatively stable and specific to AML, making it an ideal target for MRD detection [95, 96, 97]. One study found that the presence of mutant NPM1 strongly predicted recurrence in patients after two cycles of induction chemotherapy and was associated with reduced survival [98, 99]. A larger study produced similar findings, showing a 3‐year cumulative incidence of relapse (CIR) of 82% in MRD‐positive patients compared with 30% in MRD‐negative patients after two cycles of intensive chemotherapy (*p* < 0.001) [98]. Despite this, patients with persistent MRD positivity can still derive substantial therapeutic benefit from hematopoietic stem cell transplantation [100, 101].

With the growing application NGS, MRD detection via NGS has gained widespread recognition for its prognostic value [102, 103, 104, 105, 106]. A study involving 131 patients demonstrated that those with no detectable mutations had significantly better CIR and OS compared with those with residual mutations (2‐year CIR 24 vs. 46%; *p* = 0.03; 2 years OS 77 vs. 60%; *p* = 0.03) [103]. Similarly, in a larger MRD study of 482 patients involving 54 genes, mutations present during CR were associated with a higher a higher recurrence rate at 4 years (55.4 vs. 31.9% if there was no detectable mutation; *p* < 0.001), independent of variant allele frequency (VAF) [102]. MRD measured by NGS before HSCT also serves as a valuable prognostic indicator.

### 3.2 Evaluating Efficacy and Improving Prognosis

At the time of diagnosis, the treatment response is a critical predictor of disease progression in childhood AML. MRD serves as a valuable biological marker [107], evaluating the efficacy of different treatment strategies and guiding treatment decisions. Studies have demonstrated the value of MRD in assessing therapeutic effectiveness and determining the best course of treatment [108].

MRD dynamics, both before and after transplantation, have proven useful in classifying chemotherapy resistance. Patients with MRD‐negative status before and after transplantation are classified as sensitive, while those with intermediate or resistant statuses tend to experience poorer outcomes. Research shows that consolidation therapy improves prognosis, while maintenance therapy alone is considerably less effective [109, 110]. Furthermore, MRD dynamics provide more reliable prognostic information than single‐point analyses, offering insights into specific clinical questions [111].

In MM, MRD negativity has been closely linked to improved OS and PFS. The absence of detectable MRD is strongly associated with significantly better outcomes in both OS and PFS, providing scientific support for using MRD levels to approve new treatment protocols for MM patients. MRD evaluation in MM is considered in regulatory drug reviews and may be used as a surrogate marker for clinical endpoints [112, 113, 114].

Varghese et al. [115] found that MRD status within 6 months after the completion of treatment was an accurate and effective determinant of long‐term PFS and OS (*p* = 0.010). Patients who achieved MRD negativity had significantly better PFS and OS. In AML, patients with MRD‐positive results after conventional chemotherapy had shorter OS and relapse‐free survival. However, achieving MRD remission through immunotherapy significantly improved both OS and relapse‐free survival. MRD remission has different implications for patients with relapse‐prone or treatment‐resistant AML, possibly due to genetic instability and the presence of more aggressive subclones. These subclones contribute to resistance mechanisms and higher evasion rates [116, 117, 118, 119, 120, 121, 122].

Balduzzi et al. [123] measured MRD levels using qPCR t in 82 children with ALL at five specific time points, showing that 16 out of 22 patients with detectable MRD relapsed, with a relapse detection rate of 73%. The study also revealed that AML patients who eventually relapsed had MRD levels above 10 [−3] [123]. Pulsipher et al. [52] tested MRD levels using NGS in B‐cell AML patients at predetermined times and showed that only four out of 15 MRD‐positive patients did not experience a relapse (with MRD > 10^−6^), leading to a relapse detection rate of 73%. High‐throughput sequencing demonstrated a better predictive ability for MRD than FCM [124]. Bader et al. [125] performed five bone marrow MRD assessments of Ig/TCR genes using qPCR at five time points after transplantation in over 100 ALL patients, and those with MRD levels above 10^−4^ almost all experienced rapid relapse. Similarly, Pincez et al. [123] found that among 19 MRD‐positive patients who received therapeutic intervention, 13 subsequently relapsed, resulting in a relapse rate of 68%.

MRD monitoring has been proven critical, even without therapeutic intervention. Detecting MRD status before a clinical relapse becomes apparent can significantly reduce the recurrence rate. Various studies using different methodologies, such as qPCR and NGS, have demonstrated the significance of MRD in predicting relapse [125, 126].

### 3.3 Conducting Risk Grade Classification

Risk classification in leukemia has traditionally relied on clinical and biological factors, such as age at onset and white blood cell count. However, these criteria often fail to adequately differentiate risk, particularly in T‐ALL. MRD results provide a powerful tool for risk stratification, offering more refined prognostic insights [127].

Svaton et al. [22] conducted a stratified study using qPCR and NGS, showing that 76% of patients had consistent stratified results. The study assigned 193 patients to the standard‐risk group, 100 to the medium‐risk group, and 37 to the high‐risk group. Jin et al. [128] proposed a risk classification based on MRD, demonstrating its predictive value for recurrence timing. Their results showed that MRD detection after consolidation therapy was effective in determining whether patients would relapse (*p* < 0.05). In this study, patients were categorized according to different risk stratifications and dynamic changes in MRD before the first relapse. The low‐risk group had a recurrence time of 273 days, and the high‐risk group had a recurrence time of 230 days. Patients with persistent positive MRD had a median recurrence time of 130 days, while those who converted from positive to negative had a longer median time to relapse of 287 days. The negative‐positive fluctuation group showed the longest median time to recurrence at 447 days [128].

To compare recurrence times across low‐risk, intermediate‐risk and high‐risk groups, the study employed the Kruskal–Wallis test, which revealed significant differences in MRD dynamics affecting recurrence in MRD‐positive patients, reinforcing the predictive power of MRD detection in clinical prognosis.

Molecular risk classification based on MRD enhances the accuracy of stratifying patients, allowing for more precise distinctions between low‐risk and high‐risk individuals. This approach enables early intervention for high‐risk individuals, optimizing postremission treatment decisions while minimizing unnecessary interventions for patients with a lower likelihood of recurrence [89, 129, 130, 131].

### 3.4 Directing Treatment

The individualized approach to leukemia treatment increasingly relies on MRD assessment, which plays a pivotal role in personalizing treatment plans. MRD reflects the presence of leukemia cells that persist after treatment and is monitored dynamically to guide therapeutic decisions.

At the end of induction therapy, patients with MRD levels below 10^−4^ generally have a favorable prognosis and can often be treated with lower‐intensity chemotherapy regimens, reducing the risk of adverse side effects while maintaining treatment efficacy. In contrast, patients with higher MRD levels, such as those at or above 10^−2^, may require more aggressive treatments strategies, such as higher‐dose chemotherapy or hematopoietic stem cell transplantation, to manage MRD effectively. Clinical trials have demonstrated that tailoring therapy based on MRD levels can significantly improve patient outcomes, as seen in the GEM2012menos65 trial [132]. Additionally, MRD detection informs decisions on the timing of treatment modifications and the need for further interventions.

In developed countries, MRD detection technology has achieved high proficiency, especially in the clinical application of hematologic tumor diseases. For example, the Japanese Pediatric Cancer Leukemia Treatment Collaboration Group has integrated MRD detection into their treatment protocols for ALL. They adjust chemotherapy regimens and treatment timing based on MRD levels at various stages of therapy (P2, P3, P4, P5), aiming for precise, goal‐oriented treatment adjustments.

### 3.5 Assessing Risk of Recurrence after Transplantation

Accurate quantification of MRD is a crucial prognostic tool for OS and PFS following chemoimmunotherapy and allogeneic transplantation [133, 134]. MRD levels are typically used to predict relapse risk; however, the detection threshold can be influenced by several factors, including the timing of detection, the protocol used, and the sample type, leading to variability in results [135, 136].

For instance, a study on ALL patients demonstrated that none of those with negative MRD status detected by NGS relapsed over a 70‐month follow‐up period [137]. This highlights the predictive value of MRD status before transplantation. Bader et al. [138] conducted a retrospective study showing that MRD levels measured prior to stem cell transplantation were strongly correlated with recurrence risk. Patients with high, low, and negative MRD levels had 5‐year PFS rates of 28, 48, and 78%, respectively.

**FIGURE 5:**
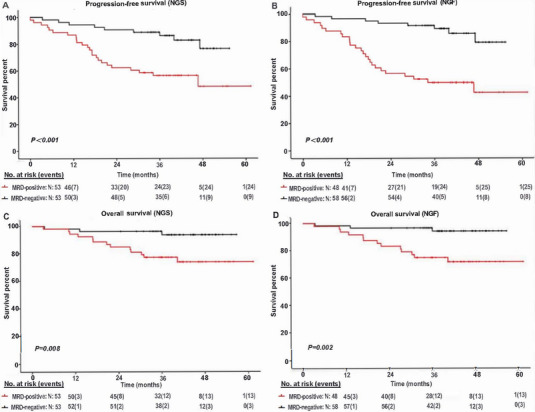
Kaplan–Meier analysis of PFS and OS of MRD‐positive versus MRD‐negative. Patients Using NGS and NGF. Kaplan–Meier curves compare progression free survival (PFS) and overall survival (OS) between MRD‐positive and MRD‐negative subsets, revealing no significant differences in detection results between NGS and NGF. The analysis demonstrates that MRD‐negative patients have significantly better PFS and OS rates than MRD‐positive patients, when time calculated from MRD assessment 3 months posttransplantation. Negative patients are indicated in black, while positive patients are shown in red, with patient risk levels detailed at each time point. Events are represented between parentheses. (A) PFS of NGS‐based results. (B) PFS of NGF‐based results. (C) OS of NGS‐based results. (D) OS of NGF‐based results. *Abbreviations*: next‐generation sequencing (NGS); next‐generation flow (NGF); minimal residual disease (MRD). Reproduced with permission from Ref. [144], Copyright © 2020, The Author(s).

Comparative studies using FCM and PCR methods have shown that MRD‐negative patients often experience significantly improved PFS compared with MRD‐positive patients across various disease conditions. According to NGS or next‐generation flow (NGF) prognostic analysis, MRD‐negative patients exhibit superior 3‐year PFS and OS compared with their MRD‐positive counterparts (Figure 5) [139].

In specific subgroups, such as transplant‐eligible newly diagnosed multiple myeloma patients and those ineligible for transplantation, MRD‐negative status has been associated with prolonged PFS. MRD‐negative CR is increasingly recognized as a strong predictor of improved OS and PFS [140, 141, 142, 143, 144, 145, 146, 147, 148, 149]. Current research emphasizes that MRD status prior to transplantation is a critical indicator in the multivariate analyses that consider first response, immune phenotype, transplant management, donor type, graft‐versus‐host disease and sex. Consistent MRD detection is indicative of increased recurrence risk and poorer disease‐free survival and OS, underscoring the importance of monitoring MRD levels at various time points [14, 150, 151, 152, 153, 154].

## 4 Challenges and Future Directions of MRD Detection

MRD has been an important biomarker for recurrence prediction and treatment selection in cancer patients. The clinical application of MRD is limited due to the low specificity and sensitivity of technologies such as FCM, PCR and NGS, as well as the biological characteristics of residual tumor cells, including antigen transfer, clonal regression, heterogeneous genome of blast cells and lack of specific targets, resulting in false positive or false negative MRD results [155]. We can start by improving the specificity and sensitivity of detection technology, pay attention to the emerging MRD detection technology, and continue to improve the existing detection technology, ultimately personalized treatment.

### 4.1 Challenges and Limitations in MRD Detection

Current MRD detection methods, while effective, face several challenges.

Although FCM is a widely used technique for MRD detection, it suffers from limited sensitivity and a lack of standardization [156, 157]. Variations in laboratory equipment, sample handling, and instrument configurations contribute to inconsistent results across different laboratories [158]. The combination of markers used for AML MRD detection varies, causing operators to rely on gating strategies. Interpretation of such test results requires a high level of expertise in MRD detection based on FCM [159, 160]. Additionally, the immunophenotype of leukemic cells can change due to subclonal evolution, complicating the detection process [156].

qPCR offers high sensitivity but provides only relative quantification of target mutations or fusion transcripts. Ct values reflect the relative copy number rather than absolute quantities, making comparisons between different assays challenging. To overcome this limitation, analysis is often performed in combination with target and standardized transcripts, making comparisons difficult [161]. Variability in qPCR results across laboratories, including issues with false positives, highlights the need for improved standardization and quality control. Scott et al. [162] sent AML samples with various MRD levels to different laboratories in different countries and regions for MRD testing and found many false positive results. Current work therefore focuses on the standardization and quality control of qPCR‐based MRD assays to generate comparable MRD results [163].

NGS is a powerful tool for detecting MRD with high sensitivity, yet it is not without limitations. Sequencing errors, including base miscalls and false positives, particularly with single nucleotide variants, can affect MRD detection accuracy [164, 165]. Despite advancements in error correction tools, achieving comprehensive and precise error removal remains a significant challenge.

### 4.2 Future Directions and Research Opportunities

#### 4.2.1 Enhancing Detection Sensitivity and Specificity

Improving the sensitivity and specificity of MRD detection is crucial. Sensitivity is influenced by the number of cells analyzed, and current protocols often do not meet the required sensitivity levels for early detection of molecular relapse [166, 167]. The commonly used MRD detection samples come from bone marrow or peripheral blood, and a large number of bone marrow aspirates are very painful and impractical for patients. In addition, the sensitivity of MRD detection is closely related to the detection protocol used, and more sensitive MRD detection methods can detect molecular relapse earlier in patients at low risk for relapse [74, 168].

High specificity is equally important to avoid false positives, which can lead to unnecessary treatment intensification and associated toxicities. NGS offers greater specificity compared with qPCR, reducing the likelihood of false positives and enabling more accurate risk stratification and treatment adjustments [53, 169]. Future MRD strategies may increasingly rely on NGS to complement or even replace traditional qPCR methods for quantification [22].

#### 4.2.2 Emerging MRD Detection Technology

##### 4.2.2.1 Combining NGS with Advanced Diagnostic Techniques

Clonal hematopoiesis is characterized by somatic mutations in hematopoietic stem cells present in the bone marrow, and patients who have undergone chemotherapy for nonhematologic malignancies have a high probability of subsequent myeloid malignancies [170, 171, 172, 173, 174, 175]. However, clonal hematopoiesis can persist after a patient has achieved CR, and at the same time it cannot represent residual cancer cells [176], which increases the difficulty of identifying leukemia cells and makes MRD detection difficult. Combining MFC with genomic and immunophenotypic analysis, such as single‐cell DNA sequencing, may enhance the ability to distinguish between clonal hematopoiesis and true leukemia [177]. This integrated approach may help facilitate the differentiation of clonal hematopoiesis‐related mutations from true leukemia mutations by NGS.

##### 4.2.2.2 Liquid Biopsy

Liquid biopsy represents a promising advancement in MRD detection by analyzing tumor components from blood samples [178, 179] and quantifying MRD in hematologic malignancies [180, 181]. This method offers the potential for noninvasive, real‐time monitoring of MRD, reducing the need for painful bone marrow aspirates. Liquid biopsies can assist in personalized treatment planning [182], predicting recurrence‐free survival [183], and evaluating treatment efficacy.

##### 4.2.2.3 Digital PCR

Digital PCR (dPCR) is an emerging technology that partitions the initial sample into numerous nanoscale PCR reactors that are further analyzed with end point PCR within the nanoscale reactors [184]. Characterized by the ability to achieve absolute quantification, coupled with very high sensitivity, dPCR offers potential for greater standardization and precision compared with traditional PCR methods and may become a valuable tool for detecting low‐frequency mutations and MRD in various cancers [25, 185, 186].

#### 4.2.3 Personalized Medicine and MRD

The advancement of MRD detection technologies is critical for personalized medicine, especially in hematological malignancies. Standardized protocols and integration of multiple technologies are essential to ensure reproducibility and maximize the clinical utility of MRD detection. Combining different techniques, such as NGS with other methods and emerging technologies, could enhance MRD detection sensitivity and specificity, leading to more personalized and effective treatment strategies [187].

## 5 Conclusion

In summary, MRD detection has become a pivotal component in clinical monitoring due to its significant role in predicting treatment outcomes and guiding therapeutic strategies [188]. This review has extensively covered various MRD detection methods, including FCM, PCR, and NGS, highlighting their principles, advantages, and limitations. It also explored the clinical applications, challenges, and future directions in MRD detection.

Currently, several methods are available for MRD detection, with NGS standing out with its exceptional sensitivity reaching up to 0.0001%. NGS excels in identifying clonal rearrangements in B and T cell antigen receptor genes, making it an invaluable tool in complementing disease treatment. Considering the challenges associated with clonal hematopoiesis, it is recommended that NGS and iRepertoire's BCR IgH PCR panel be combined to further improve MRD detection. iRepertoire's MRD panel integrates UMIs with PCR allowing each cell to be counted and eliminates PCR and sequencing error, detecting 1 in 1,000,000 cells using the RNA‐based IgH panel. Emerging technologies, such as iRepertoire's, offer promising advancements that could further refine MRD detection and contribute to personalized treatment approaches.

To address the variability in MRD detection results, there is a need for standardized protocols across different laboratories. This includes developing uniform guidelines for sample handling, assay implementation, and data interpretation to ensure consistency and comparability of MRD results.

Early‐stage MRD detection is crucial for predicting relapse and tailoring treatment strategies. Methods that offer less invasive alternatives, such as peripheral blood‐based MRD detection and liquid biopsy, should be prioritized for development and clinical adoption to improve patient comfort and monitoring efficiency.

MRD detection technologies should be incorporated into personalized medicine strategies to optimize treatment plans. By combining various detection methods and integrating them into clinical practice, healthcare providers can more accurately stratify patients, adjust treatment plans and potentially improve patient outcomes.

Continued research into emerging MRD detection technologies and their applications is essential. This includes exploring the potential of new techniques and improving existing ones to further enhance the accuracy, sensitivity, and clinical utility of MRD detection.

In clinical practice, MRD detection has become integral to evaluating cancer treatment plans and prognosis indices. Continuous MRD testing provides comprehensive clinical information, offering insights into the overall treatment effects on patients and serving as a crucial reference for disease risk classification. Early‐stage MRD detection not only reflects treatment efficacy but also plays a pivotal role in determining disease risk classification, underscoring its importance.

## Author Contributions

M. L. S. was responsible for writing the manuscript, and W. J. P. played a guiding role in reference collection and manuscript revision. X. J. Y., J. R., and C. L. T. were mainly responsible for the revision of English grammar and expression. Z. C., Z. W., and Y. D. checked different parts of the manuscript. N. Y. H., H. N. L., and S. L. provided revisions. All authors read and approved the final manuscript.

## Conflicts of Interest

The authors declare no conflicts of interest.

## Data Availability

The authors have nothing to report.
